# Supplementary material for: Ubiquitin E3 Ligase AaBre1 Responsible for H2B Monoubiquitination Is Involved in Hyphal Growth, Conidiation and Pathogenicity in Alternaria alternata
Source: Genes (Basel). 2020 Feb 21;11(2):229. doi: 10.3390/genes11020229 (PMC7074354; doi:10.3390/genes11020229)
Supplement: Supplementary file 1 [file genes-11-00229-s001.pdf]

SUPPLEMENTARY INFORMATION FOR

Ubiquitin E3 ligase AaBre1 responsible for H2B monoubiquitination involved in hyphal growth, conidiation and pathogenicity in *Alternaria alternata*

Ye Liu[1,2], Jingjing Xin[1,2], Lina Liu[1], Aiping Song[1], Yuan Liao, Zhiyong Guan[1], Weimin Fang[1], Fadi Chen[1,*]

Table S1 Primer sequences used in this study.

Primer	Sequence (5'-3')	Relevant characteristics
AaBre1-FULL-F<br>AaBre1-FULL-R	CTTCGCTGCCCTATCTACGA<br>CAAGGAAACGCAGGTGATGT	PCR primers for amplification of the full length of *AaBRE1*
AaBre1-UP-F<br>AaBre1-UP-R	TCTCTGTACTTGCAGGCGCT<br>CAAAATAGGCATTGATGTGTTGACCTCCATGTCATCTTTCCACGGCAA	PCR primers to amplify *AaBRE1* upstream fragment for the construction of *AaBRE1* deletion mutants
AaBre1-DOWN-F<br>AaBre1-DOWN-R	CTCGTCCGAGGGCAAAGGAATAGAGTAGCGCATCAATAATCTTGAAGCC<br>GCAAGTCTTGATGACCGTGTT	PCR primers to amplify *AaBRE1* downstream fragment for the construction of *AaBRE1* deletion mutants
AaBre1-KO-F<br>AaBre1-KO-R	AGGACATTCGGAGCTTCAGTG<br>ATTCGCGTTGAGCGTGTTTT	PCR primers to amplify *AaBRE1* deletion fragment for the construction of *AaBRE1* deletion mutants
AaBre1-ID-F<br>AaBre1-ID-R	TCTACGACACCCGTGTCTCCT<br>GTTTCGAATGCGAGCACAAT	PCR primers for identification of *AaBRE1* deletion transformants
AaACT-QRT-F<br>AaACT-QRT-R	CATTGGCAACGAGCGTTT<br>AGCAAGAATGGAACCACCGA	Quantitative real-time PCR primers for analysis of *AaBRE1* expression level
AaBre1-QRT-F<br>AaBre1-QRT-R	TTGGTCACCGAGAAGGATCAT<br>CGAGGTACCATTGGTGGCTT	PCR primers for amplification of the reference gene *AaACTIN* in quantitative real-time PCR assays
AaBre1-GFP-F<br>AaBre1-GFP-R	CGAATTGGGTACTCAAATTGGTTCTCTGTACTTGCAGGCGCTG<br>AACAGCTCCTCGCCCTTGCTCACTAGATGCACACGCATCGTGTC	PCR primers to amplify the native promoter region and open reading frame of *AaBRE1* for GFP fusion protein construction
Aabre1gfp-id-F<br>Aabre1gfp-id-R	GAACGCCATCACTTCCGAAG<br>TGACGGCTCAAACATGGTTG	PCR Primer for PYF11-*AaBRE1*-GFP plasmid identification

Table S2 Summary of sequencing reads after filtering.

Sample	Clean reads	Clean bases	Error rate(%)	Q20(%)	Q30(%)	GC content(%)
Aa_1	63807750	9.5E+09	0.0113	98.8	96.08	54.35
Aa_2	57760018	8.6E+09	0.0115	98.71	95.84	54.2
Aa_3	59187960	8.8E+09	0.0114	98.72	95.87	53.95
BR_1	63903526	9.5E+09	0.0115	98.72	95.85	54.24
BR_2	64455978	9.59E+09	0.0114	98.75	95.94	54.4
BR_3	61988020	9.22E+09	0.0115	98.7	95.81	54.25

Table S3 Statistics of reads mapping to Alternaria alternata genome by Hisat2.

Sample	Total reads	Total mapped	Multiple mapped	Uniquely mapped
Aa_1	63807750	53761354(84.26%)	175274(0.27%)	53586080(83.98%)
Aa_2	57760018	48556970(84.07%)	167092(0.29%)	48389878(83.78%)
Aa_3	59187960	47922431(80.97%)	160242(0.27%)	47762189(80.7%)
BR_1	63903526	54211520(84.83%)	222002(0.35%)	53989518(84.49%)
BR_2	64455978	55862624(86.67%)	204065(0.32%)	55658559(86.35%)
BR_3	61988020	53142677(85.73%)	214514(0.35%)	52928163(85.38%)
